# Brain Morphological Signatures for Chronic Pain

**DOI:** 10.1371/journal.pone.0026010

**Published:** 2011-10-13

**Authors:** Marwan N. Baliki, Thomas J. Schnitzer, William R. Bauer, A. Vania Apkarian

**Affiliations:** 1 Department of Physiology, Northwestern University, Chicago, Illinois, United States of America; 2 Department of Physical Medicine and Rehabilitation, Northwestern University, Chicago, Illinois, United States of America; 3 Department of Neuroscience, University of Toledo, Toledo, Ohio, United States of America; 4 Department of Anesthesia, Feinberg School of Medicine, Chicago, Illinois, United States of America; 5 Department of Surgery, Feinberg School of Medicine, Chicago, Illinois, United States of America; University of Cordoba, Spain

## Abstract

Chronic pain can be understood not only as an altered functional state, but also as a consequence of neuronal plasticity. Here we use *in vivo* structural MRI to compare global, local, and architectural changes in gray matter properties in patients suffering from chronic back pain (CBP), complex regional pain syndrome (CRPS) and knee osteoarthritis (OA), relative to healthy controls. We find that different chronic pain types exhibit unique anatomical ‘brain signatures’. Only the CBP group showed altered whole-brain gray matter volume, while regional gray matter density was distinct for each group. Voxel-wise comparison of gray matter density showed that the impact on the extent of chronicity of pain was localized to a common set of regions across all conditions. When gray matter density was examined for large regions approximating Brodmann areas, it exhibited unique large-scale distributed networks for each group. We derived a barcode, summarized by a single index of within-subject co-variation of gray matter density, which enabled classification of individual brains to their conditions with high accuracy. This index also enabled calculating time constants and asymptotic amplitudes for an exponential increase in brain re-organization with pain chronicity, and showed that brain reorganization with pain chronicity was 6 times slower and twice as large in CBP in comparison to CRPS. The results show an exuberance of brain anatomical reorganization peculiar to each condition and as such reflecting the unique maladaptive physiology of different types of chronic pain.

## Introduction

Increasing evidence supports the idea that chronic pain could be understood not only as an altered perceptual state, but also as a consequence of peripheral and central neuronal reorganization. Studies in animal models of chronic pain have demonstrated that sustained pain is accompanied with molecular, neuronal, and structural changes in the periphery and the spinal cord [1]. Recent anatomical and functional imaging studies in humans are beginning to provide insights into the brain reorganization associated with chronic pain. Different chronic pain conditions seem to evoke distinct brain activity patterns, which reflect not only pain but also the clinical manifestations associated with the disease [2], [3]. In addition there is accumulating evidence that chronic pain alters brain dynamics beyond pain perception by distorting spatial and temporal properties of the brain default mode network (DMN), first shown in chronic back pain (CBP) patients for an attentional task [4], and now also seen during resting state in multiple chronic pain conditions [5], [6], [7]. Moreover, chronic pain is associated with distorted information flow in brain reward/motivation circuitry [8].

Brain reorganization for chronic pain has also been investigated by comparing its morphology between chronic pain and healthy controls. Altered brain morphology was first shown for CBP patients [9], [10] and is now reported in many pain conditions, including fibromyalgia [11], [12], [13], [14], complex regional pain syndrome (CRPS) [15], osteoarthritis (OA) [16], [17], irritable bowl syndrome [18], [19], [20], headaches [11], [21], [22], [23], chronic vulvar pain [24], in females suffering from menstrual pains [25], as well as in animal models of chronic pain [26], [27]. Across the human studies the most consistent observation is regional decreases in grey matter in the pain patients (although increases and no change are also reported) and, even though many studies emphasize involvement of brain regions associated with pain processes, the data also suggests that unique brain regions are impacted in different types of chronic pain. Most importantly, it remains unclear whether morphological reorganization exclusively impacts pain processing circuitry and whether the sites of local reorganization mirror specific patterns of brain functional states seen for distinct types of chronic pain (for differing views see [2], [17], [28], [29]).

Recent studies demonstrate that many of the gray matter changes observed in pain patients subside with cessation of pain [16], [17], [30]. In addition, it has been shown that the observed morphological differences in chronic pain conditions often correlate to the number of years of pain individuals have been suffering with the condition as well as its intensity [9], [12], [15], [28]. These results suggest that the brain morphological changes may be reversible in nature and are a consequence of pain perception. However, this pain perception is embedded in a larger maladaptive context, wherein physical movement, daily function, and mood are reshaped by the presence of pain. Within this framework we argue that brain morphological changes should also reflect experience/learning dependent changes underlying the specific pathology and consequent cognitive/behavioral cost of the disease [31], [32]. However, our understanding of the extent and nature of morphological distortions and the specificity of these changes in different chronic pain conditions may be critically limited using the standard assessment methods. The majority of human brain morphometric studies are based on voxel-wise comparisons between groups. Implicit in such analyses is the assumption that expected changes are local and do not interact with each other. However, if brain morphometry is being re-carved as a consequence of coping, suffering, and related behavioral changes, then the interaction across brain regions should also be distorted with chronic pain, which can be studied when brain grey matter is viewed as an interconnected network.

Here we evaluate changes in brain structure using high-field magnetic resonance imaging (MRI) in three chronic pain patient groups: CBP, CRPS, OA, relative to healthy matched controls. The primary hypothesis tested is that distinct brain morphological changes are associated with different chronic pain types. We examine the latter at the voxel level, and more grossly when the brain is parceled into Brodmann areas-based subdivisions. We also test for the impact of extent of chronicity of pain on brain morphology by examining its influence commonly across chronic pain types. Given that different chronic pain conditions seem to underlie distinct brain functional networks and recent studies link changes in brain functional connectivity to anatomical connectivity [33], [34], we advance and test the hypothesis that the brain grey matter when viewed as a network will exhibit distinct properties for different chronic pain conditions. Moreover, we develop a novel approach for quantifying morphological changes based on an index derived from within subject co-variation of brain gray matter, and demonstrate that this approach accurately classifies individuals to their respective clinical chronic pain conditions. The latter index provides a measure with which we could also quantify the time course and amplitude for re-organization of the brain grey matter network with pain chronicity.

## Results

### Total neocortical gray matter volume is lower only in CBP

**Figure 1:**
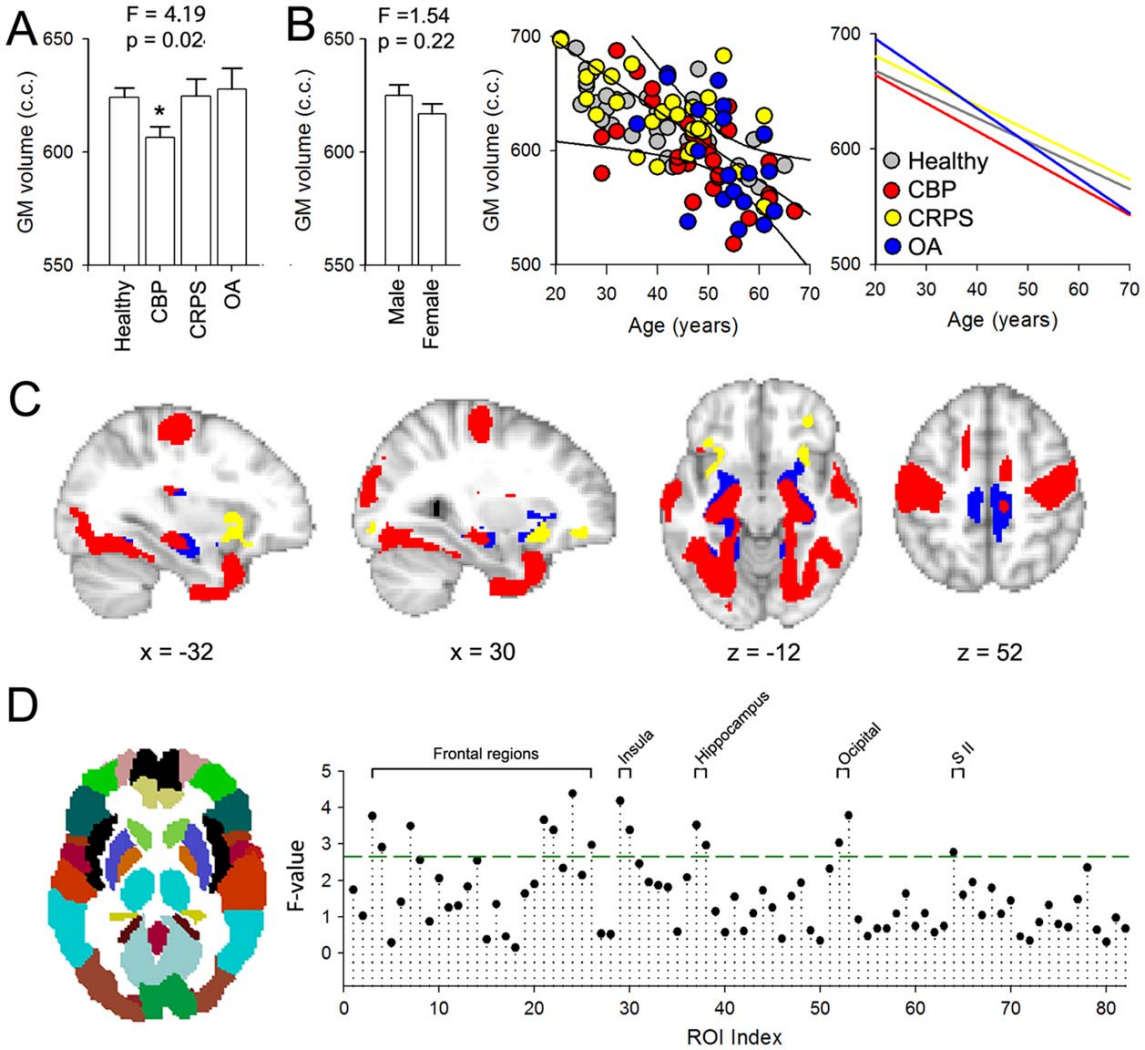
Cortical gray matter changes in three chronic pain conditions assessed at multiple scales. **A.** Average total neocortical gray matter (GM) volume for the three patient groups, CBP (n = 36), CRPS (n = 28) and OA (n = 30), and healthy controls (n  = 46). Group effect was assessed using an ANCOVA with age, gender and intracranial volume as confounds and was significant (F _(4,128)_  = 4.19, P  = 0.022). Planned pair-wise contrasts between each chronic pain group and healthy controls showed that only CBP exhibited a significant decrease in total neocortical GM volume (p<0.01). **B.** Bargraph shows that total neocortical GM volume did not differ by gender across the groups (F _(4,128)_  = 1.54, P  = 0. 22). Scatter plot presents neocortical GM volume in relation to age for each subject, color-coded by group. All patient groups and healthy controls exhibit a significant negative correlation between neocortical GM volume and age. Right panel shows the slopes computed independently for each group, which did not differ from each other. **C.** Gray matter morphological changes assessed by voxel based morphometry (VBM). The three groups of patients were contrasted separately to specifically matched healthy controls. Shown are the t-test statistics maps for patients<controls (t-score >3.0, corrected for multiple comparisons across space by permutation testing, clustering determined using TFCE). The three patient groups exhibited distinct cortical patterns of regional gray matter density decreases (CBP  = red, CRPS = yellow, OA = blue). **D.** Gray matter morphological changes assessed by ROI based GM density comparison. The cortex was subdivided into 82 predefined regions, which approximate the left and right hemisphere Broddmann areas. Gray matter density was averaged across all voxels within each ROI and compared across groups after correcting for age, gender and global brain volume. Plot shows the F-value for across group comparison, green line indicates significance threshold (F _(1,124)_>2.7). Brain regions above threshold are labeled and include multiple frontal regions, insula, secondary somatosensory cortex (SII), hippocampus, and occipital cortex.

Differentiating normal age-related changes from the effect of disease on the total brain volume may potentially provide new insights regarding mechanisms of chronic pain. Here we compare total neocortical grey matter (GM) volume between the three patient groups and controls, after correcting for intracranial volume, gender, and age. Total neocortical GM volume showed a significant difference across groups (F _(4,128)_  = 4.19; p  = 0.022) (**Figure 1A**). Post-hoc comparisons showed that only the CBP patients exhibited a significant decrease in neocortical GM volume when compared to healthy controls (p = 0.008). Total ventricular volume (sum of 3^rd^ and 4^th^ ventricle volumes) was also measured as a control and did not differ across groups (F _(4,128)_  = 1.08; p  = 0.48).

Males and females did not differ in neocortical GM volume (across all subjects) (F_(1,128)_  = 1.54; p  = 0.22) (**Figure 1B**). When we examined the relationship of total neocortical GM volume to age we observed a strong negative correlation for all groups (healthy age dependent slope: −2.01, R = 0.77, p<0.01; CBP age dependent slope: −2.43, R = 0.65, p<0.01; CRPS age dependent slope: −2.15, R = 0.64, p<0.01; OA age dependent slope: −3.00, R = 0.51, p = 0.02) (**Figure 1B**). These age-related decreases were similar for all groups and matched previous estimates [9], [15], [35], [36].

### Voxel-wise and gross regional analyses reveal specific patterns of decreased gray matter density for each chronic pain condition

VBM is a flexible voxel-wise whole-brain statistical analysis technique that can be used to assess between-group differences in local brain tissue content and to examine correlations between tissue content and other measures of interest. To identify regional increases or decreases in GM density for the different pain conditions, each group was entered as a condition into a separate model, and linear contrasts performed against age and gender matched healthy controls (unpaired t-test, t-score >3.0, corrected for multiple comparisons across space using permutation testing, clustering determined using TFCE). Only significant decreases of GM density were observed in the patients relative to healthy controls. The three VBM statistical maps show distinct regional GM density decreases for each chronic pain group (**Figure 1C** **, Figure S1).** CBP was associated with decreased GM density in bilateral posterior insula, secondary somatosensory cortices, pre- and post-central regions in addition to hippocampus and temporal lobes. CRPS showed decreased GM density primarily in the anterior insula and orbitofrontal cortex. Decreased GM density in OA was localized to portions of the insula and mid ACC in addition to the hippocampus, paracentral lobule and regions of the inferior temporal cortex (Table S1).

It is interesting to note that GM decrease in density in chronic pain patients showed distinct amounts of overlap between conditions. The OA VBM map showed 89.7% overlap with that of CBP. The CRPS was the most dissimilar of the 3 patient groups and it showed 8.7% overlap with CBP and 9.l% with OA. In addition, GM density decreases were not limited to regions that have been shown to be involved in pain perception, representation, or modulation. For example, all groups exhibited decreased GM density in the inferior temporal gyrus, and CBP and OA also showed decreased GM density in the hippocampus and visual cortex, brain regions that do not receive direct input from the ascending nociceptive pathways and are not implicated in processing painful information.

In addition to voxel-wise VBM, we examined GM density changes at a more gross level by parceling the brain to 82 predefined ROIs and computing mean GM density in each ROI, as derived from the VBM analysis. The 82 regions comprised 41 cortical regions in each hemisphere, corresponding approximately to classical Brodmann areas. Similar to the voxel-wise VBM analysis, age, gender, and total intracranial volume were regressed out. Differences in GM density across the four subject groups for all ROI-s were tested using a 1-way-ANOVA (corrected for multiple comparisons using the Holm-Bonferroni correction). Results for the comparison are shown in Figure 1D, and generally agree with the results obtained with voxel-wise VBM comparisons. Regions that best differentiated GM density between the groups included pain related areas such as secondary somatosensory cortex, bilateral insula, and dorsal and orbital fontal regions, in addition to regions not specifically related to pain processing: the hippocampus and occipital cortex.

### Relating grey matter density decreases to chronicity of pain

To elaborate on the specificity of GM density changes in relation to clinical pain parameters, we first performed a whole-brain voxel-wise correlational analysis between GM density results obtained by VBM and pain intensity, pain duration, and their interaction independently for each group. Previous reports indicated that the degree of GM density changes show a relationship with duration and intensity of pain in different pain conditions [9], [12], [15], [22]. In the present study GM density did not show any significant correlations with pain duration, intensity or their interaction for any group.

We also investigated the contribution of depression, anxiety and drug usage to GM density changes. We performed a whole-brain voxel-wise correlation analysis between GM density results obtained by VBM and Beck Depression Inventory (BDI), Beck Anxiety Inventory (BAI), and Medication Quantification Scale (MQS) scores independently for each group. GM density did not show any significant correlation with anxiety, depression or MQS. In addition the relationship between GM density and pain intensity and/or duration did not change when anxiety, depression and medication use were used as covariates of no interest.

**Figure 2:**
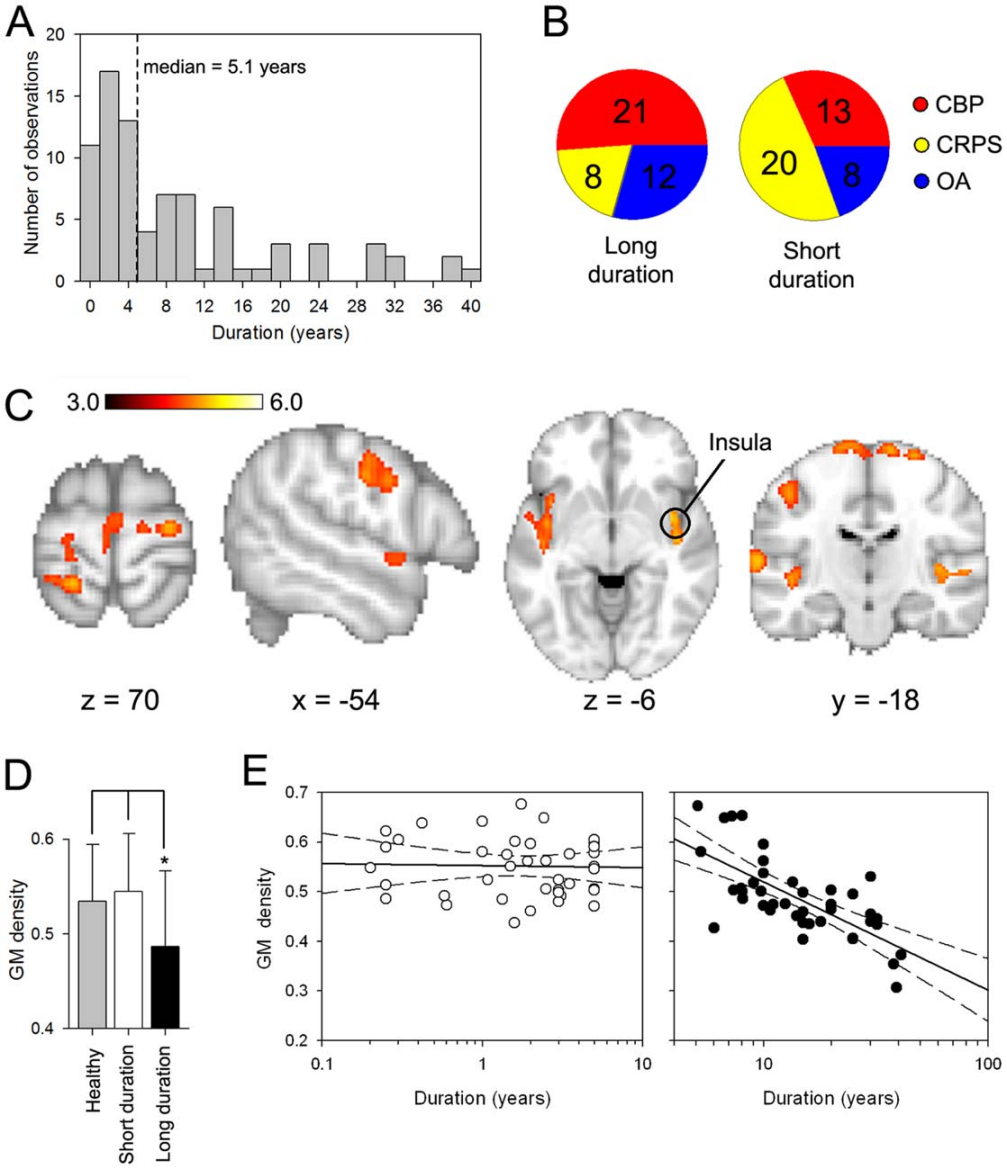
Relationship between Gray matter density and duration of chronic pain. **A)** Distribution of pain duration for all patients. Patients were divided into 2 subgroups (short duration and long duration) based on the median (median duration = 5.1 years, dashed line). **B)** Pie charts show the frequency of each patient population for the 2 subgroups. **C)** Brain regions that exhibit significant decreased GM density for long duration compared to short duration pain (voxel-wise VBM, unpaired t-test, t-score >3.0, corrected for multiple comparisons across space using permutation testing, clustering determined using TFCE). Regions that showed significant decreases in GM density for longer pain duration included primary sensory and motor regions, as well as insular cortex. **D)** Bargraph shows the mean +/- SD for GM density for the right insula for the short duration, long duration and healthy groups. Insular mean GM density was significantly less for the long duration group when compared to short duration or healthy groups. **E)** Scatter plots show the relationship between Insula GM density and duration for the short duration group (left panel, open circles) and long duration (right panel, filled circles). The insula shows a significant relationship with pain duration only in the group when pain was experienced for more than 5 years (R = 0.71, p<0.01).

In contrast, when we examined the effect of chronicity of pain irrespective of pain duration type (voxel-wise VBM contrast between short duration>long duration for all patients; separated by the median of pain duration = 5.1 years, **Figure 2A, 2B**), we observe multiple brain areas distinguishing between short and long duration chronic pain (**Figure 2C**), where we again observe only decreased GM density and only in the long duration grouping. The brain areas identified were mainly sensory and motor regions as well as bilateral insula. As a case example, and a post-hoc regional analysis, we extracted the peak GM density for the right insula and compared its values between healthy subjects, and short and long duration groupings for chronic pain patients. GM density for the insula (healthy: 0.53±0.06, short duration: 0.54±0.05, long duration: 0.47±0.06; values expressed as mean±S.D) was significantly lower in the long duration group compared to the healthy (unpaired t-test, t = −2.56, p <0.05) and short duration groups (t<−2.78, p<0.05) (**Figure 2D**). Moreover, the right insula GM mean showed a tight correlation with individual subjects' pain duration (log scale), only for the group with long duration chronic pain (R = 0.79, p<0.01; **Figure 2E**). These results, therefore, imply that living with chronic pain, independent of its type and past about 5 years, imparts GM decrease in proportion to the pain duration within a common set of brain regions.

### Chronic pain specifically disrupts whole-brain morphological structure of the brain

**Figure 3:**
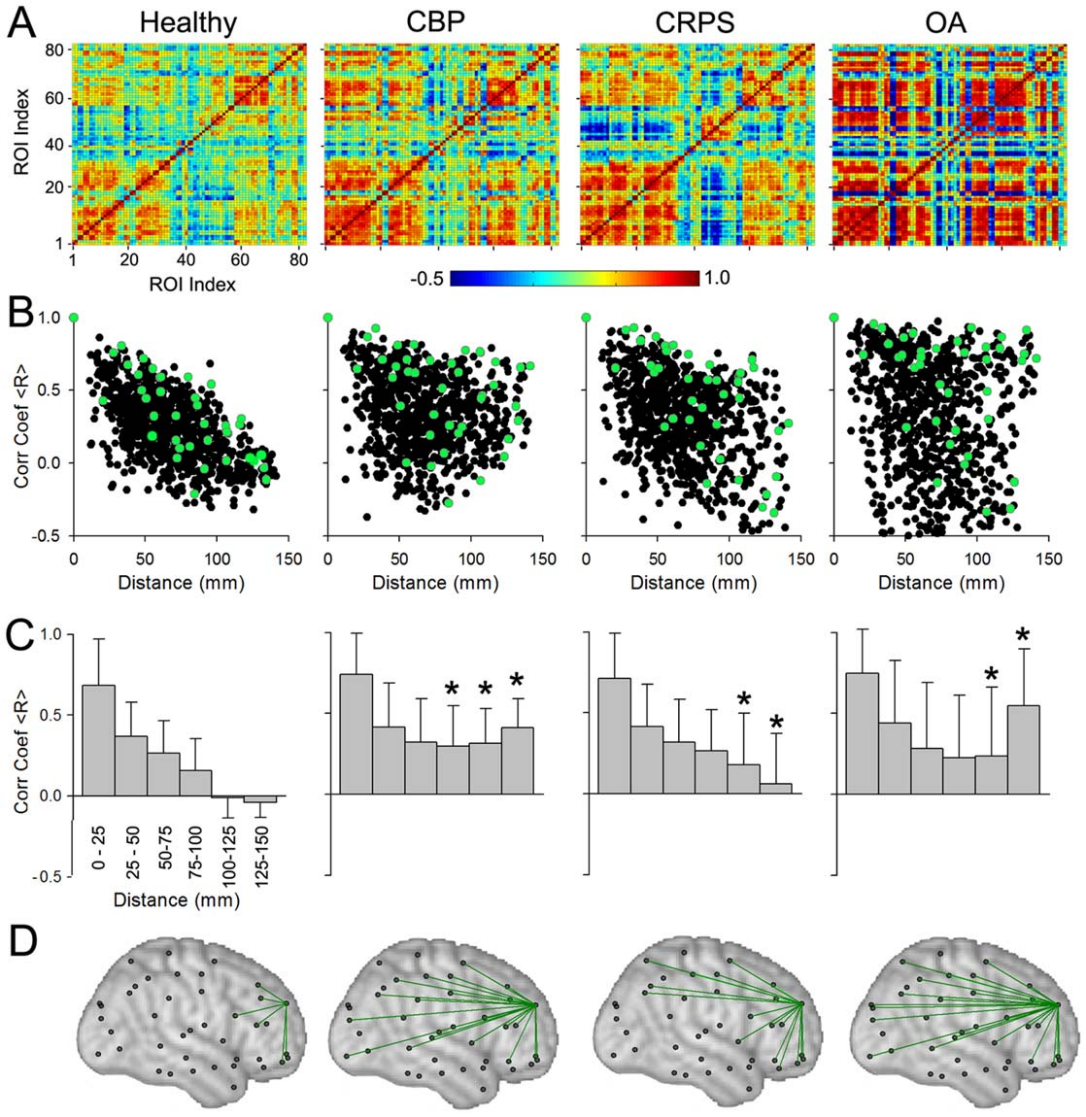
Cortical structural covariance is specific for different chronic pain groups. **A)** Structural covariance was studied by calculating pair-wise correlation of GM density between the 82 ROI-s across subjects separately for healthy controls, CBP, CRPS, and OA, after correcting for age, gender, and total intracranial volume. Resultant correlation matrix shows widespread increases in correlation strength in all three patient groups. **B)** Scatter plots show the left hemisphere pair-wise correlations (for the 41 ROIs) plotted against mean distance between pairs. Healthy subjects show a linear dependency on distance. This relationship is disrupted in unique ways in each chronic pain condition. Green dots indicate the correlation of one example region to the rest of the brain. **C)** Bar plots are the same data as in (B) after binning distances into 6 ranges, 25 mm each. Mean +/- SD for pair-wise correlation coefficients are shown for each bin, in each group. Patients show higher correlations between ROIs that are far apart (>100 mm apart; asterisks p<0.01 comparing means for each bin to its counterpart in the healthy controls). **D)** Spatial illustration of changes in correlation for a frontal cortex ROI (same area illustrated in green in **B**). Significantly strong connections (r>0.6, p<0.05) are plotted (green lines) on standard brain (black marks are centers of ROIs). Strong connectivity is observed with neighboring regions in the healthy group, while all three chronic pain patient groups show enhanced long distance connections.

Here we tackle the issue of the influence of local morphological disturbances on the inter-relationship of morphology in relation to other brain regions. Specifically we address the issue, given that the different chronic pain groups exhibit distinct regional GM density decreases, how do these local changes relate to the morphological structure of the whole cortex? Whole-brain anatomical organization can be abstracted by compiling a matrix of correlations between all pairs of regions, averaged for appropriate subject groupings. As the gross 82 ROI based VBM data dramatically compresses representational dimensionality, it can be readily used to generate such a matrix of associations between (VBM derived and corrected) gray matter density estimates for each pair of ROIs, in each group separately. Four correlation matrices for the four groups are illustrated in **Figure 3A**. Qualitatively we observe that, compared to the correlation matrix for healthy subjects (**Figure 3A, left panel**), all three patient groups show increased correlation strengths (positive and negative) for many pairs of ROI-s. To visualize the spatial properties of these correlations, we displayed the strength of the correlations as a function of the physical distance between any given ROI pair, for each hemisphere. For the left hemisphere (**Figure 2B, left panel**), healthy subjects showed a linear relationship between correlation strength and distance (R = 0.67, p<0.01). Thus, whole-brain GM density structure can be captured by this simple linear rule, which shows that ROIs that are located close to each other are more similar in GM density than those farther apart, and the decrement in GM density similarity is proportional to the distance separating the involved regions. Yet, this rule may have local exceptions, as there are large scatter of values away from the linear fit. This relationship was attenuated for CRPS (R = 0.29, p<0.05) and disturbed for CBP (R = 0.12, p = 0.18) and OA (R = 0.09, p = 0.31) (**Figure 2B** **right three panels**). Comparison of the slopes of each pain group to that of healthy controls yielded significant differences (p<0.01 for each comparison). Qualitatively we observe that the shape of the cloud of scatter of points is distinct for each chronic pain group. Within the 2-dimensional correlation-to-distance space, CBP group shows less observations in the right lower corner and increased points in the right top corner; CRPS group shows more values throughout the right half of the space; and OA group observations seem to expand and fill all quadrants of the space. These distinct patterns suggest that the whole-brain inter-relationship of GM density is uniquely shifted in each chronic pain patient group. Some of these between group structural differences can be captured quantitatively by binning the distances (**Figure 3C**, 6 bins of 25 mm each). All three patient groups show significantly higher mean correlation (mean of coefficients for all voxels within bin) compared to controls for ROI-s that are physically separated by 100, 125 and 150 mm. In addition OA patients exhibit the most variability in connectivity strength for any distance (size of SD).

To illustrate the global disruption of correlations across the brain, we examined a single (arbitrarily chosen) frontal ROI and its strength of connectivity across the four groups. The distribution of correlation values for this ROI closely mimic that seen across all ROIs for each grouping (**Figure 3B**). By applying a significance threshold (correlation values R>0.6, corrected for multiple comparisons using Holm-Bonferonni) we can observe the spatial distribution of the strongest correlations between GM density in this region and the rest of the brain. **Figure 3D** illustrates these connections on the left hemisphere, where again we observe strengthening of long distance correlations in the three chronic pain groups in contrast to the healthy subjects in which case strong correlations are localized to the neighborhood of the ROI.

### Subject classification based on gross brain morphology

**Figure 4:**
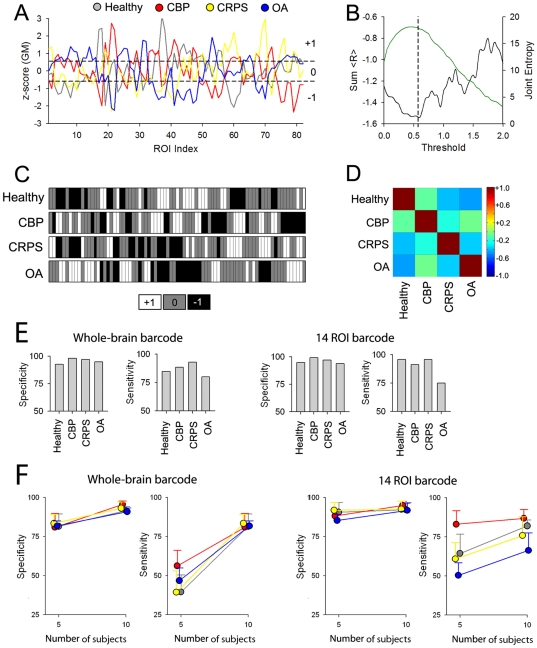
The ROI based GM density profile can be transformed into a barcode and used to classify chronic pain patients with high accuracy. A) The GM density for each ROI was normalized within subject and averaged for each group. The four groups show distinct variations of GM across the brain. Dashed lines are thresholds implemented to tag ROIs to three different classes, high (+1), average (0), or low (−1) GM values. B) Threshold was selected to optimize the difference between groups. Black trace shows the relationship between the sum of R (the sum of the pair-wise correlations coefficients between the barcodes for the 4 groups) and threshold implemented to generate the bar graphs. The green trace represents the amount of information measured as joint entropy for the 4 groups as a function of threshold. The 4 groups showed the most difference at a threshold of 0.56 (vertical dashed line). C) Group barcodes derived for the data in (A) using the selected threshold. D) Pair-wise correlations across the four groups for the selected threshold. All between groups correlations are negative. E) Sensitivity and specificity of correctly identifying individual subjects to their respective group based on the maximum correlation of each barcode with group barcodes. Left panel is when the whole-brain barcode, shown in C, is used. Right panel is the result when 14 ROIs that best discriminate between the groups (shown in Figure 1C) were used. The procedure shows very high specificity and sensitivity, as random classification would correspond to 25% sensitivity and specificity. F) Dependence of sensitivity and specificity on number of brains used to calculate average barcodes. Whole-brain or 14 ROI-based barcodes derived from a subset of subjects per group (5 and 10 respectively) and classification applied to the rest of the subjects. Shown are mean±S.D. of specificity and sensitivity from 10 iterations for random choices of 5 and 10 subject group barcodes. Specificity exhibited robust results for both tests, while sensitivity seemed to be more dependent on the number of subjects used and the specific group tested.

Using three different approaches above we examined the impact of chronic pain conditions on cortical morphology. Sum total of these results suggest that the anatomy of the brain in chronic pain is reorganized distinctly from that of healthy subjects, and in a pattern unique for each of the pain conditions studied. The latter implies that brain morphology may provide the means by which individuals can be diagnostically classified to their appropriate grouping. Here we devise a novel approach to identify a ‘brain signature’ for the different conditions, and test its accuracy in properly classifying individual brains. As the whole-brain structural properties seem distinct for each chronic pain group, we used GM density across all 82 ROI-s as the vector defining each individual. **Figure 4A** shows the group average normalized GM density for patients and healthy controls. Differences in the shape of these vectors reflect average group brain regional distinctions of GM density, which emphasizes within subject co-variation of GM density. To further simplify this representation, these vectors were binned into three categories (high GM density = +1, average GM density = 0, and low GM density = -1). The threshold was selected to optimize differences between groups while retaining the maximum amount of information (**Figures 4B**). This approach emphasizes divergence from mean by marking peak changes and thus reducing noise. The resultant ‘barcodes’ for the 4 groups are displayed in **Figure 4C**. The pairwise correlations of the group barcodes with each other are shown in **Figure 4D**. All groups exhibited negative correlations with each other, with OA and healthy being the most different (R = −0.61) and OA and CBP the most similar (R = −0.23). Individual subject barcodes were generated with the same approach. Calculating correlations between each subject's barcode to the 4 group barcodes assessed the similarity of each subject's brain morphology to the four groups. The highest correlation coefficient was used to classify any given subject to the corresponding category (Healthy, CBP, CRPS or OA). Using this method we were able to classify with high specificity (Healthy: 86.2%, CBP: 94.2%, CRPS: 92.2%, and OA: 90.9%) and sensitivity (Healthy: 80.0%, CBP: 81.2%, CRPS: 90.5% and OA: 72.6%). We also tested two other classification techniques, logistic regression and artificial neural networks, and these resulted in similar performance accuracies as the simple maximum correlation method (data not shown).

We also tested classification using a more restricted barcode by including only ROI-s that exhibited largest differences in GM density across groups (14 ROI-s that passed threshold, **Figure 1D**). Using this 14 ROI barcode we were able to predict each class with specificity (Healthy: 94.8%, CBP: 92.0%, CRPS: 96.9% and OA: 94.9%) and sensitivity (Healthy: 95.7%, CBP: 91.2%, CRPS: 94.1% and OA: 75.0%) (**Figure 4E**). The 14 ROI based classification improves mainly sensitivity relative to the whole-brain based results.

As the available data set remains relatively small, the above classifications were based on average barcodes calculated across all members of each group and then testing membership for each individual. Thus it suffers from the bias of double dipping. To overcome this bias we also tested classification, for the whole-brain and 14 ROI barcodes, when subsets of subjects (5 and 10) were used to determine the group barcode. The subset of subjects was selected per group at random to generate the group barcode and the sensitivity and specificity were computed using the rest of subjects. This process was iterated 10 times resulting in mean+/- S.D. sensitivity and specificity values for each condition and subset size. Group average specificity and sensitivity values are shown in **Figure 4D**. When the number of subjects was restricted to 5 to generate group barcodes, for the whole-brain barcode we were able to predict classes with high specificity (Healthy: 81.6±8.0%, CBP: 80.8±8.9%, CRPS: 82.6±5.8%, and OA: 81.5±3.3%) and sensitivity (Healthy: 39.6±10.9%, CBP: 56.2±9.9%, CRPS: 38.6±13.8% and OA: 46.5±8.2%), and for the 14 ROI barcode we obtained better specificity (Healthy: 90.6±6.3%, CBP: 88.1±2.5%, CRPS: 91.0±5.1%, and OA: 85.0±4.0%) and sensitivity (Healthy: 64.35±12.45%, CBP: 82.94±8.67%, CRPS: 60.00±10.53% and OA: 50.00±7.55%). When 10 subjects were used to generate the group barcodes, for the whole brain barcode we were able to predict the class with higher specificity (Healthy: 91.7±2.6%, CBP: 95.4±2.3%, CRPS: 92.3±4.0% and OA: 90.6±2.3%) and sensitivity (Healthy: 77.2±6.1%, CBP: 82.4±6.0%, CRPS: 78.2±6.2% and OA: 69.0±8.8%), and for the 14 ROI barcode we did not improve specificity (Healthy: 92.2±4.0%, CBP: 94.9±2.8%, CRPS: 91.7±5.2% and OA: 91.5±4.9%) or sensitivity (Healthy: 81.95±3.97%, CBP: 86.94±5.58%, CRPS: 75.00±12.14% and OA: 66.00±11.25%) (**Figure4F**). Overall, by increasing the number of subjects (from 5 to 10) used to generate the average barcode we improve accuracy of classification for both whole-brain and 14 ROI based barcodes, and performance becomes similar to that obtained when barcodes are derived from the complete data set. Interestingly, whole-brain barcode performs better than 14 ROI based barcode when the group averages are derived from the larger number of subjects. The obtained accuracy of the approach is remarkable even when very small numbers of subjects are used to generate the group barcodes, attesting to the robust and homogeneous differences in brain morphology between the groups.

**Figure 5:**
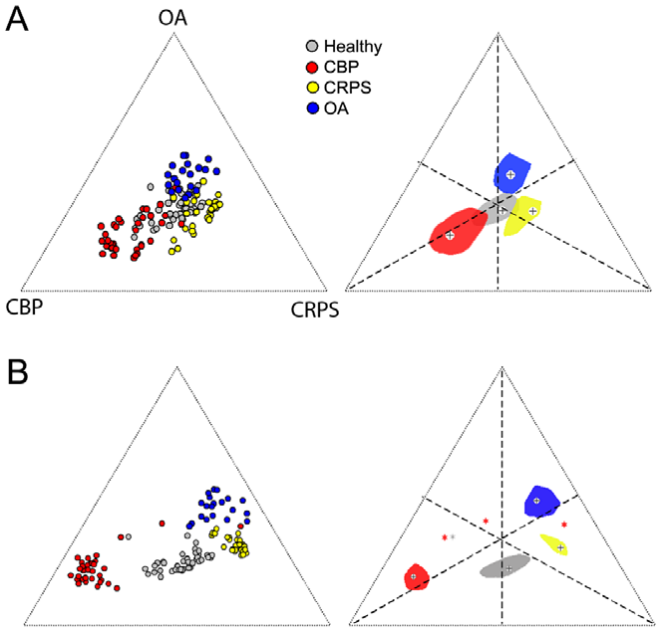
Individual brain and group GM interrelationships based on barcodes. **A)** Whole-brain group-averaged barcodes (Figure 4 C, D, E) were used to localize the relative relationship between individual brains (left), and group averages (right). **B)** Same as A except the barcode is derived from the 14 ROI barcodes (Figure 4 F). The distance from the three poles and from the center (corresponding to healthy controls) was computed from the correlation of individual subject barcode with the 4 group barcodes. Left panels localize individual brains relative to the center (healthy controls) and poles of the equilateral triangle defining the three patient groups. Right panels show the bi-median (cross) and 2-dimensional inter-quartile distances (color contours) of each group relative to the center and poles of the triangle. Different colors represent different groups. Outliers are shown as stars (three CBP and one healthy control brains).

To visualize the extent of the similarities and overlap between groups and individuals, we computed a distance (d) between each subject's barcode and the 4 group barcodes, based on the correlation coefficients. **Figure 5** shows the relative distribution of all subjects and the median and interquartile contours for each group, for the whole-brain (**Figure 5A**) and 14 ROI barcodes (**Figure 5B**). The groups are far more distinct for the 14 ROI barcodes. This segregation implies that if we use a more complex classification algorithm based on subdividing this 2-dimensional space then we should be able to classify individual brains at even a higher accuracy than shown above. This Figure also quantifies the relative distances of brain morphology between groups and individuals, and shows that CBP brains are most dissimilar to healthy subjects' brains, followed by OA, while CRPS brains have the shortest distance to healthy subjects' brains. Additionally, CBP brains are far distant from CRPS or OA, and CRPS and OA are much closer to each other than CBP brains.

### Relating whole-brain morphological reorganization to chronicity of pain

**Figure 6:**
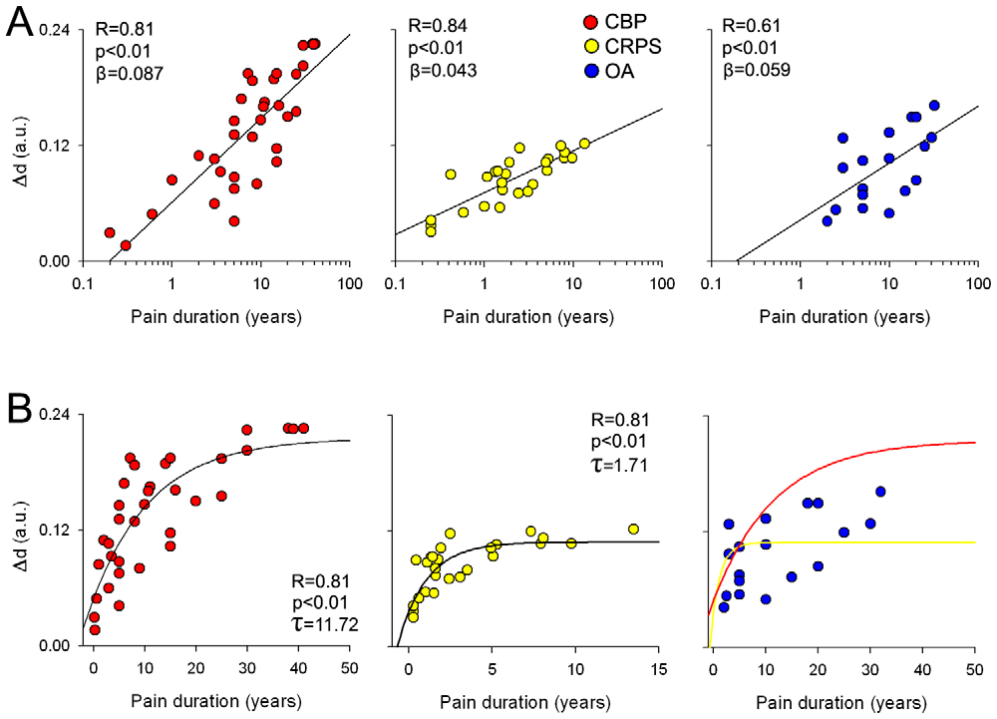
Relating whole-brain GM reorganization to pain chronicity. **A)** Scatter plots show the relationship between relative bar-code distances, Δd, for individual patients and duration of chronic pain in CBP (red), CRPS (yellow) and OA (blue). The relative distance was computed as the Euclidean distance between individual patients' location in the ternary plot from the center for the mean of healthy controls (Figure 5A), thus reflecting extent of global gray matter reorganization, where larger distances indicate larger deviations form healthy. All patient groups exhibited a significant correlation between the distance and duration of pain in log scale. **B)** Same data in A plotted as a linear function of pain duration. Distance relationship to pain duration followed an exponential growth function in CBP (left plot, red circles) and CRPS (middle plot, yellow circles), but not in OA (right) plot. Yellow and red traces in right plot show the best fitted curves for CBP and CRPS respectively.

Here we investigate the relationship of the observed whole-brain morphological changes to clinical characteristics. The relative distance, Δd, for each individual patient from the mean coordinates of healthy subjects (see **Figure 5A**) was computed and submitted for correlation analysis with pain duration, intensity and depression for each patient group independently. We found that Δd, which signifies the degree of deviation of whole-brain morphological co-variation from norm, is significantly related to pain duration in all patient groups. **Figure 6A** shows the relationship of Δd and duration of chronic pain in log scale. The CRPS group showed the highest correlation (R = 0.84, p<0.01) followed by CBP (R = 0.81, p<0.01) and OA (R = 0.64, p<0.04). When we compare the slopes between groups, only the CBP and CRPS showed a significant difference (CBP β = 0.087, CRPS β = 0.043, t-score = 4.12, p<0.01). These results indicate that pain duration and whole-brain gray matter reorganization are interrelated, for durations spanning from 3 months to 42 years, for all patient groups. In order to elucidate the temporal evolution of this relationship, we investigate the linear relationship between Δd and pain chronicity. We found that it significantly followed an exponential growth function in CBP (R = 0.82, p<0.01) and CRPS (R = 0.81, p<0.01), but not in OA (exponential and linear fits do not differ in OA, yet it seems to have characteristics in between CBP and CRPS) (**Figure 6B**). In addition the time constant for the CBP was significantly larger in CBP compared to CRPS (CBP τ = 11.72 years, CRPS τ = 1.71 years, t = 6.30, p<0.01). These results directly link gross whole-brain reorganization to the main clinical parameter, its chronicity, and indicate that the deviation of the GM network from the normal brain is about twice as large and 6 times slower in CBP than in CRPS. It is worthy to note that Δd did not show any significant relationships with pain intensity or depression.

## Discussion

Our results show that different clinical chronic pain conditions are associated with unique brain morphological changes, both locally and grossly when GM is viewed as an interconnected network. In addition, the impact of extent of chronicity of pain imparts regional voxel-wise decreases in GM shared across the groups investigated, and yet reorganizes the GM as a network to distinct amounts and at distinct rates in some of the groups studied. We utilized different approaches to quantify and compare structural changes at different spatial scales: 1) we assessed whole-brain cortical volume to examine global changes in brain structure. This parameter was only reduced in CBP and is consistent with our previous reports in which we observed decreased whole-brain volume in CBP [9] but not in CRPS [15]. 2) We used voxel-based morphometry (VBM) to assess localized changes in GM density and observed only regional GM decreases, which were more similar between CBP and OA, and distinct in CRPS. 3) When VBM data was contrasted between long and short duration chronic pain groupings, we observe somatosensory, insular and motor regions exhibiting decreased GM and specifically show that the insula GM decreases with a characteristic time constant only when pain is maintained for >5 years. 4) We parceled the brain to 82 brain regions approximating Brodmann Areas and generated a structural covariance to map anatomical interrelationships within the cortex for each group. The latter analysis showed that the OA brain is the most disrupted, and that all three pain patient groups' brains show distinct patterns of reorganization relative to healthy controls. 5) We used the 82-region parcellation to generate a barcode and demonstrated that this measure can robustly classify individuals to their respective groupings, and this approach demonstrated that CBP brain as a co-varying network is most distinct from healthy subjects while OA and CRPS are more similar to each other and to healthy brains. 6) Finally, the barcode analysis also led to quantifying the extent and rate of change of the brain GM network with pain chronicity.

Whole-brain VBM, where GM density is contrasted between subject groups for each GM voxel, remains the most commonly used tool to assess brain morphological changes in humans. Since the first report of brain grey matter distortions in chronic back pain [9], a rapidly accumulating literature has been documenting evidence for brain morphological changes in diverse clinical pain conditions (see above), all of which are based on voxel-wise comparisons of either GM density or GM thickness. Here, using voxel-wise VBM contrasts, we observe that all three patient groups showed decreases in regional GM density compared to respective matched healthy controls. The CBP group showed the most spatially extended pattern. The OA patients exhibited voxel-wise decreased GM density in many of the same regions as in CBP, and the OA results agree with earlier observation regarding GM decreases in the hippocampus [17], and insula and S2 [16]. Decreased gray matter in CRPS was restricted to anterior insula and parts of the orbital frontal cortex, closely replicating our previous observations [15]. Although a few studies have reported regional increases in GM in some pain conditions [24], [37], [38], the most common observation has been decreased GM density or thickness, and the present results confirm the latter for three chronic pain conditions.

Do chronic pain patients have a common brain GM distortion signature? Based on the results of a meta-analysis of VBM changes in 12 different patient cohorts, May [39] argues that chronic pain conditions are associated with structural changes within a common set of brain regions involved in pain perception and modulation, with the cingulate and insular cortices exhibiting most consistent decreased GM density in the cohort. Evidence for this claim is supported partially by the results in this study. For example all groups exhibited decreased GM in the insular cortex. In addition, the CBP and OA groups exhibited similar GM density decreases especially in the cingulum, S1, and S2 cortices. Furthermore duration of chronicity of pain, examined across all patient populations, was associated with additional GM decreases in brain regions known to receive nociceptive inputs and involved in pain perception and modulation, namely insula, S1 and S2 [2], [3]. However, despite these similarities, our data also showed non-overlapping GM density decreases in diverse brain regions. Indeed, brain areas that exhibited the most change in GM density across the three patient groups included the hippocampus, multiple lateral frontal regions and portions of the occipital lobe, demonstrating that morphometric changes are not limited to pain specific regions, but may reflect specific behavior and physiological changes associated with prolonged coping with persisting pain.

If, instead of using voxel-based contrast, we parcel the brain into 82 ROI-s and normalize ROI GM values relative to within subject variability, then fourteen gross brain regions distinguish between the four groups. Many of these ROIs coincide with the regions identified by voxel-wise contrasts. This approach allows building a group specific barcode, which enables using within subject variability of gross GM values as a biomarker of chronic pain. We demonstrate that, using whole-brain or 14-ROI based barcodes we can accurately classify subjects to their respective groups, and localize individual brains in relation to the group and in relation to other groups. This approach has the potential of being used in comparing between different disease states and treatments, especially in chronic pain where large co-morbidities are commonly assumed [40], as well as within and across various neurodegenerative conditions.

The reduced dimensionality of the ROI based analysis provides the means for examining across regional co-variations, a method adapted from recent techniques used to analyze network properties of GM [34], [41], [42]. With this approach we reveal that the brain GM as a network shows a simple linear distance dependence in healthy controls. To our knowledge, this is the first demonstration that gross BA-based brain network organization tightly reflects the distance separating pairs of BA-s from each other, and this dependence is most likely a reflection of genetic and developmental competitive processes underlying maturation of the cortical mantle. The current study is also the first to examine anatomical GM networks in chronic pain. All patient groups exhibited changes in inter-regional connectivities compared to healthy controls, and uniquely distorted distance dependencies, especially between non-adjacent regions (more than 100 mm apart). The observed increases in GM density across non-adjacent regions may reflect physiological changes in wide-brain distributed functional networks. This is supported by recent evidence which showed that in healthy subjects nodes within each functional network exhibited tightly correlated gray matter volumes [41].

Similar whole-brain reorganization was recently demonstrated using a very different approach: the linear relationship between white-matter anisotropy to total gray matter volume seen in healthy subjects is disrupted in CRPS patients [15]. Recent evidence shows that functional specialization can lead to related anatomical changes and regions that are anatomically connected exhibit strong correlations in cortical thickness [42], perhaps because connectivity confers a mutually trophic effect on reorganization of connected regions, based on Hebbian plasticity rules [43], [44]. Moreover, whole-brain anatomical networks seem to possess a degree of organization consistent with brain functional networks [33], [34]. Furthermore, such anatomical networks exhibit specific reorganization in schizophrenia (Bassett et al., 2008) and neurodegenerative diseases [41]. As we demonstrate that the gross GM network reorganization has characteristic time constants, distinct patterns of reorganization, and distinct magnitudes of deviation from the normal brain, we conclude that each condition uniquely impacts on the brain. As we observe that regional decrease in GM density, for example in the insula, also exhibits a characteristic time constant (slope of log-linear plot), we infer that the whole-brain time constants should reflect anatomical and functional reorganization for local changes as well as related functional and anatomical connectivity changes, each of which most likely possesses specific time constants. Thus, we can conclude that living with distinct chronic pain conditions confers a brain anatomical network reorganization, reflecting at least in part a physiological reorganization, that must be the result of the sum total of experiential changes (coping and suffering that result in novel associations and deficits in learning, in memories and valuation [45], and in extinction of memories [2], [31]), as well as peripheral and spinal cord reorganization observed in animal models [1]. Therefore, we conclude that GM distortions measured locally or as a network can be considered not only as a robust biomarker for chronic pain but also a specific biomarker that differentiates between kinds of chronic pain, and as such may be useful in disentangling between co-morbidities. Given that the anatomy of the brain is uniquely re-organized for distinct chronic pain conditions, one has to assume that the observed shifts reflect unique cognitive abnormalities, which may be unraveled based on the properties of the GM distortions. We have intentionally not pursued the inter-regional connectivity differences between the conditions studied, yet such contrasts would provide leads as to underlying cognitive/emotional/sensory distinctions, as well as suggest mechanistic differences regarding cortical plasticity for various chronic pain conditions.

### Technical issues

A notable concern is the reproducibility of VBM results for the same patient population, within and across studies. The CBP patients in this study showed a more spatially diverse pattern of GM decreases compared to our previous report [9] and those from another study[10]. We presume this difference is a consequence of multiple small incremental improvements: higher strength magnet, larger sample size, better quality control for acquired MRIs, better matching of templates, corrections for confounds and other small changes in analysis. Multiple investigators have examined brain morphometry in fibromyalgia patients [11], [12], [13], [14], [46], [47]. Most show a regional decrease in grey matter density [12], [46], [47], one shows an increase [11], and yet another finds no change at all [13]. Such variability of outcomes in a single chronic pain condition is unlikely to be a reflection of the patients studied. Instead it is more probably because of the fact that morphometric studies are complicated and subtle technical differences can bias outcomes, such as integrity of acquired MRIs, inadequate registration and/or segmentation, and choice of statistical test (parametric or non-parametric). We assume that the method we advance here, based on parceling the brain to 82 ROI-s, will be more readily usable across labs and might result in more reproducible outcomes, as the technique may not even need high resolution MRIs and has the potential of using MR images acquired in different labs and with different scan parameters, all of this, however, remains to be tested in the future.

An additional limitation of the study is the fact that chronic pain patients use various analgesic drugs over many years, which might confound observed brain morphological changes, as suggested earlier [9], [10] [15]. We quantified drug consumption using a validated questionnaire [48], which reduces drugs used for different durations and doses to a single scalar. This allowed us to examine the effect of medication on grey matter changes using a covariate analysis. No significant relationships in GM density with drug use were observed.

### Conclusions

We demonstrate that brain GM properties are distinct in three chronic pain patient conditions at multiple spatial scales, locally and globally: whole-brain, the brain parceled to 82 GM regions, the brain as a network of 82 GM regions, and voxel-wise GM. The regional GM values show highly specific group differences, enabling classification of individuals to their conditions with high accuracy. Additionally the impact of the extent of chronicity of pain can be observed commonly for all conditions and as characteristic time constants for reorganizing the brain GM network. Specific distorted GM networks for each chronic pain group studied suggests that the reorganization is related to the disease properties and as such reflects the maladaptive physiology of chronic pain. The profusion of brain anatomical reorganization with chronic pain suggests that such conditions can be used as a tool for studying large-scale brain plasticity mechanisms in general. Given the diverse spatial and temporal evidence presented here and shown in animal models [26], [27] for brain anatomical reorganization with chronic pain, a multiplicity of mechanisms should underlie these changes.

## Methods

### Subjects

The pool of subjects that participated in this study included 46 healthy subjects (26 females, 20 males; age: mean = 38.77, s.d. = 12.50 years), 36 CBP patients (13 females, 23 males; average age: mean = 48.20, s.d. = 11.38 years), 28 CRPS patients (24 females, 4 males; average age: mean = 40.57, s.d. = 7.4 years) and 20 OA patients (4 females, 16 males; average age: mean = 53.50, s.d. = 7.4 years). All procedures for this study were reviewed and approved by Northwestern University ethics and IRB committee. All participants reviewed and signed written consent forms approved by Northwestern University ethics and IRB committee. All participants were right-handed. Healthy subjects, CBP and OA patients were recruited by newspaper ads in Chicago area, whereas CRPS patients were recruited from local clinics in Chicago and a clinic in Toledo, OH. All patients were diagnosed by a clinician and fulfilled the International Association for the Study of Pain (IASP) criteria, and had to satisfy a specific list of inclusion/exclusion criteria. Patients were excluded if they reported other chronic painful conditions, systemic disease, history of head injury or coma, or psychiatric diseases. Depression is a common comorbidity of chronic pain. Therefore, patients reporting more than mild to moderate depression, as defined by Beck's Depression Inventory (BDI, global score >19) were also excluded. No cutoff threshold was used for anxiety.

### Pain parameters and medication

All patients completed the short-form of the McGill Pain Questionnaire (SF-MPQ) which includes a visual analog scale (VAS) (0 = no pain, 100 = maximum imaginable pain) and pain duration. Depression scores for all subjects that participated in the study were assessed using BDI. All questioners were given 1 hour prior to brain scanning. Drug consumption was quantified using the Medication Quantification Scale (MQS) [48], which reduces drugs used for different durations and doses to a single scalar. The clinical and demographic data, as well as pain-related parameters for the CBP, CRPS and OA patients are presented in Table S2.

### Scanning parameters

We used a 3T scanner (Siemens, Germany) to acquire high-resolution T1-anatomical brain images. For all participants, MPRAGE type T1-anatomical brain images were acquired using the following parameters: voxel size 1×1×1 mm; TR, 2500 ms; TE, 3.36 ms; flip angle = 9°; in-plane matrix resolution, 256×256; slices, 160; field of view, 256 mm.

### Total gray matter volume estimation

All subjects were included for this analysis. The T1-anatomical brain images were used to calculate cortical gray matter volume, with skull normalized to a standard brain (to compensate for body-mass variations), excluding the cerebellum, deep gray matter and brainstem. T1-anatomical brain images were also used to calculate skull-normalized lateral ventricular volumes (3^rd^ and 4^th^ ventricles), using an in-house-made mask for this purpose. SIENAX, (http://www.fmrib.ox.ac.uk/fsl/), was used to yield estimates of volumes of interest [49], [50], after automated brain extraction and tissue segmentation. Statistical analysis to compare group differences in total GM volume across groups was performed using an ANCOVA in which the effects of intracranial volume, age and gender were regressed out as covariates of no interest.

### Voxel based morphometry (VBM)

Regional gray matter density was assessed with VBM using the optimized method and nonparametric statistical contrasts [35], [51]. The FSL 4.0 software was used for brain extraction [50] and segmentation [52], and FMRIB's Linear Image Registration Tool (FLIRT) to spatially register the native images. The protocol included the following steps: first, a left-right symmetric study-specific gray matter template was built from 80 gray-matter-segmented native images (20 images were randomly selected from each group to minimize size of population bias) and their respective mirror images that were all affine-registered to a standard gray matter template (ICBM-152). The gray matter volume images were then linearly normalized onto this template. Finally, images were smoothed with isotropic Gaussian kernel (sigma = 3.5, FWHM = 8 mm). Subcortical regions, including the bilateral thalamus and basal ganglia were excluded from the VBM and subsequent analyses using an in-house-made mask since VBM can overestimate periventricular volume loss due to spatial registration errors [41], [53].

To identify gray matter differences associated with each type of chronic pain, each group was entered as a condition into a separate model, and linear contrasts were performed against age and gender matched healthy controls selected from the healthy subject data pool. Significant changes in gray matter density were assessed using permutation-based inference [54] to allow rigorous comparisons of significance within the framework of the general linear model with p<0.01. Group differences were tested against 5000 random permutations, which inherently and exactly accounts for multiple comparisons. Age, gender and total intracranial volume were used as variables of no interest. Group contrast clusters were identified using threshold-free cluster enhancement (TFCE) method, which bypasses the arbitrary threshold necessary in methods that use voxel-based thresholding and is more sensitive and interpretable than cluster-based thresholding methods [55].

### Regional based gray matter morphometry

We calculated GM density for 82 cortical regions in each participant. Regions were defined anatomically by previous template images (Pick-Atlas, Advanced Neuroscience Imaging Research Core, http://www.fmri.wfubmc.edu; MRIcro, http://www.sph.s.c.edu/comd/rorden/mricro.html) that were registered with the gray matter volume maps by an affine registration. The 82 regions compromised 41 regions in each hemisphere approximating classical Brodmann areas in addition to the hippocampus and amygdala. A list of the regions is presented in Table S3. The mean GM density for each ROI was computed as the sum of GM density of all voxels within the ROI from the VBM maps. We used linear regression to model the effects of age gender, and total intracranial volume on the full set of individual GM density measurements for each region. The residuals of this regression, which represent the GM density measurements corrected for age, gender, and total GM density, were used to compare group differences for GM density for each ROI using 1-way ANOVA.

### Covariate analysis (Inter-regional correlations)

We used Pearson correlation as the metric of association between corrected GM density estimates for each possible pair of the 82 regions in each group separately. For each pair of brain regions *i* and *j*, we computed the correlation in corrected GM density over subjects. This resulted in a 82×82 correlation matrix representing the specific associations in corrected GM density between all possible pairs of regions for each group. We also investigated the relationship between the GM density association and distance for all pair of regions. The physical distance between a pair of regions *i* and *j* was calculated as the Euclidean distance: √ ((x*_i_*-x*_j_*)^2^ + (y*_i_*-y*_j_*)^2^ + (z*_i_*-z*_j_*)^2^), where x, y and z represent the coordinates of the center of gravity of each ROI in standard space.

### Barcodes

Individual subject whole-brain barcodes were generated using the corrected GM density across all 82 ROIs as a vector. First, the GM density for each ROI was normalized for each subject (z transformation to reflect the deviation of GM density for a given ROI from the subject's mean GM density). The resultant vector was binned into three categories using high and low thresholds (high GM density = +1, average GM density = 0, and low GM density = −1). Threshold was calculated by maximizing mutual information based on joint entropy (using Mutual Info 0.9 package in Matlab). Group whole-brain barcodes for each condition (Healthy, CBP, CRPS and OA) were generated in a similar fashion using the mean GM density for each ROI per condition. We also generated 14 ROI-based barcode using the regions that showed significant differences in GM density across all groups. Group barcodes were either generated from the mean GM density of ROI-s using all subjects in each population, or from a subgroup (n  = 5 and n = 10) selected at random from the pool of subjects. To classify individuals into groups, individual barcodes were correlated to the group barcodes resulting in 4 group specific Pearson correlation coefficients. Individual subjects' brains were then classified based on the highest correlation coefficient.

### Distance plots

To visualize the extent of similarities and overlap between groups, individual subjects' barcode based distances were plotted in a Cartesian space with coordinates (x*_i_*, y*_i_*), where xi = x*_cbp_*.d*_cbp_*+ x*_crps_*.d*_crps_* + x*_oa_*.d*_oa_*+x*_normals_*.d*_normals_*)/(d*_cbp_* + d*_crps_* + d*_oa_* + d*_normals_*) and y*_i_* = y*_cbp_*.d*_cbp_*+ y*_crps_*.d*_crps_* + y*_oa_*.d*_oa_* +y*_normals_*.d*_normals_*)/ (d*_cbp_* + d*_crps_* + d*_oa_* + d*_normals_*). Where x*_cbp_* y*_cbp_*, x*_crps_* y*_crps_*, x*_oa_* y*_oa_* and x*_normals_* y*_normals_* were predetermined values and represent the coordinates of the 3 poles of an equilateral triangle and its center respectively. d*_cbp_*, d*_crps,_* d*_oa_* and d*_normals_* represent the distance of any given individual barcode from the respective group barcode and was derived from the Pearson correlation coefficient of individual and group barcodes (with spatial transformation d = (r+1)/2).

To determine the median and interquartile distances for each group in the 2-dimensional space defined by the equilateral triangle, we used the bagplot function (Matlab), which is a bivariate generalization of the univariate boxplot where the half-space location of a point is computed relative to a bivariate dataset (an extension of the univariate concept of rank) [56].

To examine the relationship between the barcode and clinical characteristics, we measured relative distance between individual patient brains and the mean location for healthy subjects, Δd. These values were correlated to pain chronicity in log scale, as well as fitted to an exponential growth curve, Δd = Δdo+Δd1(1-exp(-t/τ)), where t = duration of chronic pain, τ is characteristic time constant, and Δd1 is the asymptotic maximum deviation from healthy subjects. Their relationship was also examined for pain intensity and depression outcomes.

## Supporting Information

Figure S1 **Decreased Gray matter density in patients.** Detailed maps for gray matter morphological changes assessed by voxel based shown in Figure 1b. Red-yellow regions represent areas that exhibited significant decrease in GM density for each chronic pain condition compared to healthy. List of Foci are presented in Table S1.
(TIF)

Table S1 **Peak foci for decreased GM density in patients.** List of brain regions that exhibited significant decrease in gray matter density in patients compared to healthy. R = right; L = left; H = hemisphere; BA = Broddmann area; Sup = superior; Inf = inferior; Mid = middle. Ant  = anterior. Coordinated in mm.
(DOC)

Table S2 **Demographic and pain clinical parameters in patients.** Listed are the pain, mood and demographic data for patients participated in the study. VAS = visual analogue scale; BDI  = Beck's depression inventory, BAI = Beck's anxiety inventory. MQS = medical quantification scale. The VAS was computed from the McGill short-form questionnaire (sf-MPQ).
(DOC)

Table S3 **List of the ROIs for the automated parcellation.** Listed are the names of regions and standard space coordinates of the center. Coordinated in mm.
(DOC)
