# Supplementary figures and images for: Brain Morphological Signatures for Chronic Pain
Source: PLoS One. 2011 Oct 13;6(10):e26010. doi: 10.1371/journal.pone.0026010 (PMC3192794; doi:10.1371/journal.pone.0026010)

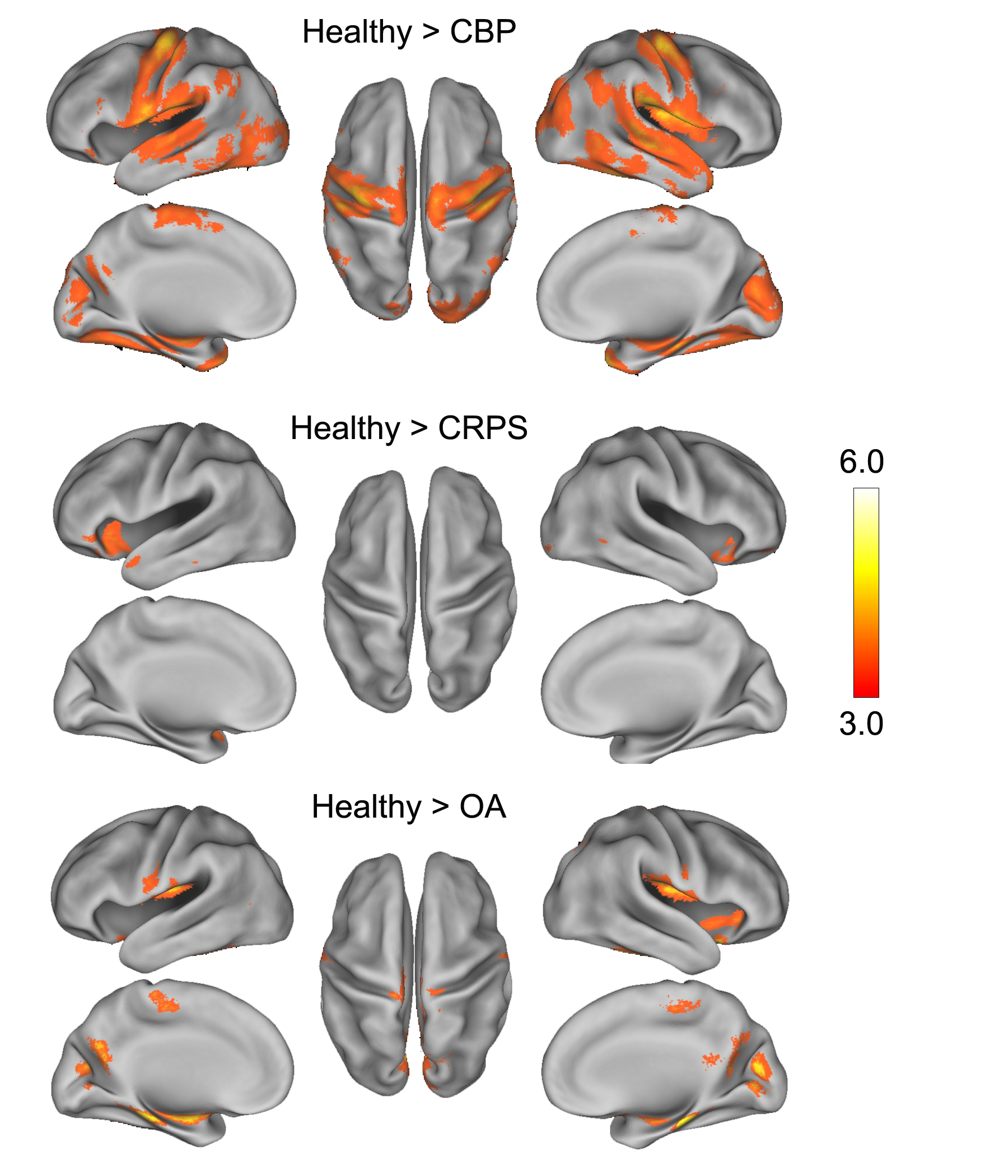

Supplement: Figure S1 — Decreased Gray matter density in patients. Detailed maps for gray matter morphological changes assessed by voxel based shown in Figure 1b. Red-yellow regions represent areas that exhibited significant decrease in GM density for each chronic pain condition compared to healthy. List of Foci are presented in Table S1. (TIF) [file pone.0026010.s001.tif]
