# Supplementary material for: Brain Morphological Signatures for Chronic Pain
Source: PLoS One. 2011 Oct 13;6(10):e26010. doi: 10.1371/journal.pone.0026010 (PMC3192794; doi:10.1371/journal.pone.0026010)
Supplement: Table S1 — Peak foci for decreased GM density in patients. List of brain regions that exhibited significant decrease in gray matter density in patients compared to healthy. R = right; L = left; H = hemisphere; BA = Broddmann area; Sup = superior; Inf = inferior; Mid = middle. Ant = anterior. Coordinated in mm. (DOC) [file pone.0026010.s002.doc]

| CBP | | | | | | |
| --- | --- | --- | --- | --- | --- | --- |
| **Brain region** | **H** | **BA** | **Coordinates**  **x y z** | | | **z-score** |
| **S2 /post insula** | L | 48 | -40 | -16 | 20 | 5.78 |
|  | R | 48 | 38 | -14 | 20 | 6.54 |
| **Precentral** | L | 6 | -36 | -20 | 64 | 5.11 |
|  | R | 6 | 36 | -20 | 56 | 5.09 |
| **Postcentral** | R | 3 | -50 | -24 | 54 | 4.36 |
|  | L | 4 | 44 | -22 | 44 | 4.40 |
| **Middle temporal** | R | 21 | 50 | -32 | 0 | 4.71 |
|  | L | 22 | -64 | -32 | 0 | 4.90 |
| **Paracentral lobule** | R | 4 | 8 | -22 | 70 | 6.87 |
|  | L | 4 | -8 | -24 | 68 | 5.23 |
| **Hippocampus** | R | 20 | 16 | -10 | -14 | 5.34 |
|  | L | 20 | -20 | -14 | -14 | 5.40 |
| **Inf temporal** | L | 20 | -48 | -36 | -18 | 4.59 |
|  | R | 20 | 46 | -22 | -24 | 4.63 |
| **Sup parietal** | R | 7 | 28 | -62 | 50 | 5.87 |
|  | L | 7 | -26 | -74 | 46 | 4.02 |
| **Fusiform** | R | 19 | 24 | -60 | -10 | 4.30 |
|  | L | 19 | -38 | -62 | 12 | 4.50 |
| **Cuneus** |  |  | -16 | -64 | 26 | 5.53 |
| **Mid occipital** | R |  | 5 | -82 | 12 | 4.30 |
|  | L |  | -3 | -82 | 10 | 4.45 |

| CRPS | | | | | | |
| --- | --- | --- | --- | --- | --- | --- |
| **Brain region** | **H** | **BA** | **Coordinates**  **x y z** | | | **z-score** |
| **Ant insula** | R | 48 | 30 | 20 | -8 | 3.54 |
|  | L |  | -34 | 18 | -4 | 4.32 |
| **Inf frontal orbital** | R | 38 | 30 | 44 | -10 | 4.50 |
| **Inf temporal** | R | 20 | 50 | -26 | -16 | 3.20 |
|  | L | 20 | -60 | -32 | -16 | 3.47 |

| **OA** | | | | | | |
| --- | --- | --- | --- | --- | --- | --- |
| **Brain region** | **H** | **BA** | **Coordinates**  **x y z** | | | **z-score** |
| **S2 /post insula** | L | 48 | -42 | -18 | 20 | 6.09 |
|  | R | 48 | 38 | -14 | 20 | 7.96 |
| **Hippocampus** | R | 20 | 30 | -16 | -12 | 6.22 |
|  | L | 20 | -30 | -18 | -12 | 6.60 |
| **Ant insula** | R | 48 | 36 | 12 | 0 | 3.51 |
| **Paracentral lobule** | R |  | 4 | -24 | 52 | 6.70 |
| **Mid Cingulum** | L |  | -10 | -24 | 48 | 7.50 |
| **Mid occipital** |  |  | 0 | -80 | 12 | 6.30 |
