# Supplementary material for: Brain Morphological Signatures for Chronic Pain
Source: PLoS One. 2011 Oct 13;6(10):e26010. doi: 10.1371/journal.pone.0026010 (PMC3192794; doi:10.1371/journal.pone.0026010)
Supplement: Table S2 — Demographic and pain clinical parameters in patients. Listed are the pain, mood and demographic data for patients participated in the study. VAS = visual analogue scale; BDI = Beck's depression inventory, BAI = Beck's anxiety inventory. MQS = medical quantification scale. The VAS was computed from the McGill short-form questionnaire (sf-MPQ). (DOC) [file pone.0026010.s003.doc]

| **CBP** | | | | | | | |
| --- | --- | --- | --- | --- | --- | --- | --- |
| **Pat** | **Age** | **Gender** | **Duration** | **VAS** | **BDI** | **BAI** | **MQS** |
| 1 | 36 | M | 15 | 3.1 | 15 | 1 | 6.8 |
| 2 | 51 | F | 0.6 | 4.8 | 1 | 2 | 4.6 |
| 3 | 47 | F | 8 | 8.0 | 12 | 7 | 0 |
| 4 | 62 | M | 38 | 5.0 | 13 | 7 | 0 |
| 5 | 49 | F | 14 | 3.7 | 8 | 13 | NA |
| 6 | 44 | M | 5 | 5.8 | 6 | 22 | NA |
| 7 | 44 | F | 5 | 5.6 | 4 | 11 | 0 |
| 8 | 66 | M | 4.6 | 6.0 | 5 | 5 | 0 |
| 9 | 24 | M | 7.7 | 7.1 | 7 | 11 | NA |
| 10 | 29 | M | 0.3 | 2.9 | 4 | 11 | 0 |
| 11 | 50 | M | 30 | 5.5 | 9 | 28 | 0 |
| 12 | 54 | M | 30 | 0.4 | 1 | 1 | 0 |
| 13 | 51 | M | 3.5 | 6.8 | 3 | 0 | 0 |
| 14 | 67 | M | 10 | 7.6 | 3 | 1 | 3.4 |
| 15 | 52 | M | 5 | 0.4 | 5 | 31 | 8.7 |
| 16 | 62 | M | 25 | 1.8 | 5 | 15 | 4.6 |
| 17 | 43 | F | 15 | 8.2 | 13 | 13 | 0 |
| 18 | 46 | M | 11 | 5.8 | 4 | 14 | 0 |
| 19 | 49 | M | 41 | 4.7 | 6 | 5 | NA |
| 20 | 67 | M | 39 | 3.5 | 16 | 9 | 6.8 |
| 21 | 39 | F | 5 | 8.4 | 5 | 19 | 0 |
| 22 | 46 | F | 16 | 5.2 | 10 | 10 | 11.2 |
| 23 | 54 | F | 1 | NA | NA | NA | NA |
| 24 | 47 | M | 5 | NA | NA | NA | NA |
| 25 | 26 | F | 3 | 6.6 | 7 | 22 | 0 |
| 26 | 31 | F | 9 | 7.9 | 2 | 9 | 21.4 |
| 27 | 32 | M | 6 | 9.1 | 4 | 27 | 0 |
| 28 | 62 | F | 2 | 1.1 | 10 | 28 | 13.6 |
| 29 | 59 | M | 10.7 | 0.3 | 4 | 4 | 0 |
| 30 | 62 | M | 25 | 1.8 | 3 | 6 | 8.8 |
| 31 | 39 | F | 7.2 | 4.3 | 11 | 19 | 15.7 |
| 32 | 44 | M | 15 | 8.4 | 5 | 15 | 7.4 |
| 33 | 57 | M | 8 | 3.1 | 7 | 11 | 0 |
| 34 | 47 | M | 20 | 8.4 | 2 | 8 | 4 |
| 35 | 52 | F | 0.2 | 7.6 | 10 | 5 | 0 |
| 36 | 44 | M | 3 | 8.6 | 6 | 9 | 0 |

| **CRPS** | | | | | | | |
| --- | --- | --- | --- | --- | --- | --- | --- |
| **Patient** | **Age** | **Gender** | VAS | **Duration** | **BDI** | **BAI** | **MQS** |
| 1 | 21 | F | 4.4 | 5.25 | 10 | NA | 26 |
| 2 | 25 | F | 6.3 | 1.92 | 13 | 12 | 47.6 |
| 3 | 26 | F | NA | NA | NA | NA | 0 |
| 4 | 26 | F | 2.2 | 0.42 | 13 | 3 | 14.7 |
| 5 | 26 | F | 4.3 | 1 | 8 | 20 | 0 |
| 6 | 28 | F | 9.8 | 2.5 | 27 | NA | 19.3 |
| 7 | 28 | F | 2.3 | 1.6 | 7 | 8 | 0 |
| 8 | 31 | F | 3.8 | 0.25 | 5 | 0 | 34.5 |
| 9 | 32 | F | 6.1 | 1.75 | 5 | 1 | 9.3 |
| 10 | 35 | F | 2.5 | 2.42 | 17 | NA | 62.2 |
| 11 | 39 | F | 6.5 | 1.33 | 9 | 2 | 8 |
| 12 | 40 | F | 8.3 | 13.5 | 10 | 14 | 54.9 |
| 13 | 41 | F | 8.9 | 3.5 | 12 | 9 | 27.5 |
| 14 | 43 | F | 5.6 | 5.08 | 14 | 17 | 36.3 |
| 15 | 44 | F | 5.9 | 1.08 | 1 | 0 | 35.6 |
| 16 | 46 | F | 5.4 | 1.58 | 11 | 5 | 6.6 |
| 17 | 47 | F | 8.2 | 7.92 | 15 | 0 | 24.5 |
| 18 | 47 | F | 5.2 | 8.08 | 3 | 4 | 29 |
| 19 | 47 | F | 7.1 | 7.33 | 13 | 5 | 15.6 |
| 20 | 49 | F | 7.5 | 4.92 | 20 | NA | 25.6 |
| 21 | 50 | F | 6.6 | 3.08 | 12 | NA | 44 |
| 22 | 50 | F | 7.7 | 1.5 | 5 | 10 | 0 |
| 23 | 53 | F | 8.2 | 0.25 | 5 | 0 | 0 |
| 24 | 61 | F | 6.2 | 0.25 | 6 | 6 | 16.8 |
| 25 | 36 | M | 2.5 | 0.58 | 6 | 24 | 0 |
| 26 | 48 | M | 2.2 | 1.42 | 13 | 0 | 8.5 |
| 27 | 56 | M | 5.6 | 0.25 | 12 | 11 | 0 |
| 28 | 61 | M | 4.4 | 9.75 | 4 | 14 | 0 |

| **OA** | | | | | | | |
| --- | --- | --- | --- | --- | --- | --- | --- |
| **Pat** | **Age** | **Gender** | **VAS** | **Duration** | **BDI** | **BAI** | **MQS** |
| 1 | 61 | M | 4.0 | 30 | 9 | 2 | 0 |
| 2 | 36 | M | 7.1 | 5 | 4 | 3 | 0 |
| 3 | 61 | M | 6.0 | 3 | 5 | 0 | 0 |
| 4 | 62 | M | 6.5 | 5 | 6 | 12 | 8.7 |
| 5 | 58 | M | 7.5 | 5 | 8 | 7 | 4.2 |
| 6 | 56 | M | 4.8 | 25 | 11 | 7 | 0 |
| 7 | 48 | F | 6.4 | 11.7 | 0 | 3 | 0 |
| 8 | 63 | M | 7.0 | 2.5 | 4 | 5 | 0 |
| 9 | 53 | M | 3.0 | 10 | 7 | 3 | 0 |
| 10 | 61 | F | 6.4 | 3 | 13 | 12 | 9.5 |
| 11 | 53 | M | 8.5 | 20 | 0 | 0 | 0 |
| 12 | 53 | M | 7.0 | 15 | 4 | 2 | 0 |
| 13 | 52 | M | 8.0 | 2 | 4 | 0 | 0 |
| 14 | 55 | M | 2.0 | 18 | 8 | 5 | 13.7 |
| 15 | 48 | M | 4.9 | 32 | 3 | 0 | 5 |
| 16 | 57 | F | 7.1 | 5 | 2 | 13 | 0 |
| 17 | 42 | M | 3.7 | NA | 0 | 5 | 0 |
| 18 | 46 | M | 3.6 | 10 | 13 | 15 | NA |
| 19 | 42 | M | 7.0 | 10 | 6 | 10 | NA |
| 20 | 54 | F | 6.0 | 20 | 7 | 11 | NA |
