# Supplementary material for: Brain Morphological Signatures for Chronic Pain
Source: PLoS One. 2011 Oct 13;6(10):e26010. doi: 10.1371/journal.pone.0026010 (PMC3192794; doi:10.1371/journal.pone.0026010)
Supplement: Table S3 — List of the ROIs for the automated parcellation. Listed are the names of regions and standard space coordinates of the center. Coordinated in mm. (DOC) [file pone.0026010.s004.doc]

| **ROI**  **Index** | **Region** | **x** | **y** | **z** | **ROI**  **Index** | **Region** | **x** | **y** | **z** |
| --- | --- | --- | --- | --- | --- | --- | --- | --- | --- |
| **1** | Precentral_L | -39 | -7 | 50 | **42** | Amygdala_R | 27 | -1 | -19 |
| **2** | Precentral_R | 41 | -10 | 51 | **43** | Calcarine_L | -7 | -80 | 5 |
| **3** | Frontal_Sup_L | -19 | 33 | 41 | **44** | Calcarine_R | 16 | -74 | 8 |
| **4** | Frontal_Sup_R | 22 | 30 | 43 | **45** | Cuneus_L | -6 | -81 | 26 |
| **5** | Frontal_Sup_Orb_L | -17 | 46 | -15 | **46** | Cuneus_R | 13 | -81 | 27 |
| **6** | Frontal_Sup_Orb_R | 18 | 47 | -15 | **47** | Lingual_L | -15 | -69 | -6 |
| **7** | Frontal_Mid_L | -34 | 31 | 34 | **48** | Lingual_R | 16 | -68 | -5 |
| **8** | Frontal_Mid_R | 37 | 32 | 33 | **49** | Occipital_Sup_L | -17 | -86 | 27 |
| **9** | Frontal_Mid_Orb_L | -31 | 49 | -11 | **50** | Occipital_Sup_R | 24 | -82 | 29 |
| **10** | Frontal_Mid_Orb_R | 33 | 51 | -12 | **51** | Occipital_Mid_L | -33 | -82 | 15 |
| **11** | Frontal_Inf_Oper_L | -49 | 11 | 18 | **52** | Occipital_Mid_R | 37 | -81 | 18 |
| **12** | Frontal_Inf_Oper_R | 50 | 14 | 20 | **53** | Occipital_Inf_L | -36 | -80 | -9 |
| **13** | Frontal_Inf_Tri_L | -46 | 29 | 13 | **54** | Occipital_Inf_R | 38 | -83 | -9 |
| **14** | Frontal_Inf_Tri_R | 50 | 29 | 13 | **55** | Fusiform_L | -31 | -41 | -22 |
| **15** | Frontal_Inf_Orb_L | -36 | 29 | -13 | **56** | Fusiform_R | 34 | -40 | -22 |
| **16** | Frontal_Inf_Orb_R | 41 | 31 | -13 | **57** | Postcentral_L | -43 | -24 | 47 |
| **17** | Rolandic_Oper_L | -47 | -10 | 13 | **58** | Postcentral_R | 41 | -27 | 51 |
| **18** | Rolandic_Oper_R | 52 | -8 | 13 | **59** | Parietal_Sup_L | -24 | -61 | 58 |
| **19** | Supp_Motor_Area_L | -6 | 4 | 60 | **60** | Parietal_Sup_R | 26 | -60 | 61 |
| **20** | Supp_Motor_Area_R | 8 | -1 | 61 | **61** | Parietal_Inf_L | -43 | -47 | 45 |
| **21** | Olfactory_L | -8 | 14 | -13 | **62** | Parietal_Inf_R | 46 | -48 | 48 |
| **22** | Olfactory_R | 10 | 15 | -13 | **63** | SupraMarginal_L | -56 | -35 | 29 |
| **23** | Frontal_Sup_Medial_L | -5 | 48 | 30 | **64** | SupraMarginal_R | 57 | -33 | 33 |
| **24** | Frontal_Sup_Medial_R | 9 | 50 | 29 | **65** | Angular_L | -44 | -62 | 34 |
| **25** | Frontal_Med_Orb_L | -5 | 53 | -9 | **66** | Angular_R | 45 | -61 | 37 |
| **26** | Frontal_Med_Orb_R | 8 | 50 | -9 | **67** | Precuneus_L | -8 | -57 | 47 |
| **27** | Rectus_L | -5 | 36 | -20 | **68** | Precuneus_R | 10 | -57 | 42 |
| **28** | Rectus_R | 8 | 34 | -19 | **69** | Paracentral_Lobule_L | -8 | -27 | 69 |
| **29** | Insula_L | -35 | 5 | 2 | **70** | Paracentral_Lobule_R | 7 | -33 | 67 |
| **30** | Insula_R | 39 | 5 | 1 | **71** | Heschl_L | -42 | -20 | 9 |
| **31** | Cingulum_Ant_L | -4 | 34 | 13 | **72** | Heschl_R | 46 | -18 | 9 |
| **32** | Cingulum_Ant_R | 8 | 36 | 14 | **73** | Temporal_Sup_L | -53 | -22 | 6 |
| **33** | Cingulum_Mid_L | -6 | -16 | 40 | **74** | Temporal_Sup_R | 58 | -23 | 5 |
| **34** | Cingulum_Mid_R | 8 | -10 | 38 | **75** | Temporal_Pole_Sup_L | -40 | 14 | -21 |
| **35** | Cingulum_Post_L | -5 | -44 | 23 | **76** | Temporal_Pole_Sup_R | 48 | 13 | -18 |
| **36** | Cingulum_Post_R | 7 | -43 | 20 | **77** | Temporal_Mid_L | -56 | -35 | -4 |
| **37** | Hippocampus_L | -25 | -22 | -11 | **78** | Temporal_Mid_R | 57 | -39 | -3 |
| **38** | Hippocampus_R | 29 | -21 | -12 | **79** | Temporal_Pole_Mid_L | -37 | 13 | -35 |
| **39** | ParaHippocampal_L | -21 | -17 | -22 | **80** | Temporal_Pole_Mid_R | 44 | 13 | -34 |
| **40** | ParaHippocampal_R | 25 | -16 | -22 | **81** | Temporal_Inf_L | -50 | -29 | -25 |
| **41** | Amygdala_L | -24 | -2 | -18 | **82** | Temporal_Inf_R | 53 | -32 | -24 |
